# Two novel cases further expand the phenotype of *TOR1AIP1*-associated nuclear envelopathies

**DOI:** 10.1007/s00439-019-02105-6

**Published:** 2020-02-13

**Authors:** Ivana Lessel, Mei-Jan Chen, Sabine Lüttgen, Florian Arndt, Sigrid Fuchs, Stefanie Meien, Holger Thiele, Julie R. Jones, Brandon R. Shaw, David K. Crossman, Peter Nürnberg, Bruce R. Korf, Christian Kubisch, Davor Lessel

**Affiliations:** 1 Institute of Human Genetics, University Medical Center Hamburg-Eppendorf, Martinistrasse 52, 20246 Hamburg, Germany; 2 Department of Genetics, University of Alabama at Birmingham, Birmingham, AL 36394 USA; 3 Department for Pediatric Cardiology, University Heart Center Hamburg, University Medical Center Hamburg-Eppendorf, 20246 Hamburg, Germany; 4 Cologne Center for Genomics, University of Cologne, 50931 Cologne, Germany; 5 Molecular Diagnostic Laboratory, Greenwood Genetic Center, Greenwood, SC 29646 USA; 6 Center for Molecular Medicine Cologne, University of Cologne, 50931 Cologne, Germany; 7 Cologne Excellence Cluster on Cellular Stress Responses in Aging-Associated Diseases, University of Cologne, 50931 Cologne, Germany

## Abstract

Biallelic variants in *TOR1AIP1*, encoding the integral nuclear membrane protein LAP1 (lamina-associated polypeptide 1) with two functional isoforms LAP1B and LAP1C, have initially been linked to muscular dystrophies with variable cardiac and neurological impairment. Furthermore, a recurrent homozygous nonsense alteration, resulting in loss of both LAP1 isoforms, was identified in seven likely related individuals affected by multisystem anomalies with progeroid-like appearance and lethality within the 1st decade of life. Here, we have identified compound heterozygosity in *TOR1AIP1* affecting both LAP1 isoforms in two unrelated individuals affected by congenital bilateral hearing loss, ventricular septal defect, bilateral cataracts, mild to moderate developmental delay, microcephaly, mandibular hypoplasia, short stature, progressive muscular atrophy, joint contractures and severe chronic heart failure, with much longer survival. Cellular characterization of primary fibroblasts of one affected individual revealed absence of both LAP1B and LAP1C, constitutively low lamin A/C levels, aberrant nuclear morphology including nuclear cytoplasmic channels, and premature senescence, comparable to findings in other progeroid forms of nuclear envelopathies. We additionally observed an abnormal activation of the extracellular signal-regulated kinase 1/2 (ERK 1/2). Ectopic expression of wild-type *TOR1AIP1* mitigated these cellular phenotypes, providing further evidence for the causal role of identified genetic variants. Altogether, we thus further expand the *TOR1AIP1*-associated phenotype by identifying individuals with biallelic loss-of-function variants who survived beyond the 1st decade of life and reveal novel molecular consequences underlying the *TOR1AIP1*-associated disorders.

**Electronic supplementary material:**

The online version of this article (10.1007/s00439-019-02105-6) contains supplementary material, which is available to authorized users.

## Introduction

Nuclear envelopathies, caused by pathogenic variants in genes that encode proteins of the nuclear envelope, are a group of genetic disorders with remarkable clinical and genetic heterogeneity (Nagano and Arahata 2000). Several such genes have been connected with human diseases displaying a wide variety of clinical symptoms, including muscular dystrophies, cardiomyopathies, lipodystrophies, skeletal anomalies, anomalies of the skin, cataracts, neuropathies, leukodystrophies, Pelger–Huet anomaly, and progeroid features. Examples include *LMNA* (MIM: 150330) (Alastalo et al. 2015; Bonne et al. 1999; Cao and Hegele 2000; Chen et al. 2003; De Sandre-Giovannoli et al. 2002, 2003; Eriksson et al. 2003; Fatkin et al. 1999; Hisama et al. 2011; Moulson et al. 2007; Muchir et al. 2000; Novelli et al. 2002; Quijano-Roy et al. 2008; Saha et al. 2010), *EMD* (MIM: 3003849) (Bione et al. 1994), *LBR* (MIM: 600024) (Hoffmann et al. 2002), *LMNB1* (MIM: 150340) (Padiath et al. 2006), *SYNE1* (MIM: 608441) (Gros-Louis et al. 2007; Zhang et al. 2007), *SYNE2* (MIM: 608442) (Zhang et al. 2007), *ZMPSTE24* (MIM: 606480) (Agarwal et al. 2003; Navarro et al. 2004), *LEMD2* (MIM: 616312) (Boone et al. 2016; Marbach et al. 2019), *LEMD3* (MIM: 607844) (Hellemans et al. 2004), and *BANF1* (MIM: 603811) (Puente et al. 2011).

The most heterogeneous group among nuclear envelopathies is due to mutations in *LMNA,* encoding A and C lamins. Alterations in *LMNA* can result in Emery–Dreifuss muscular dystrophy (MIM: 616516 and 181350) (Bonne et al. 1999), limb girdle muscular dystrophy 1B (MIM: 159001) (Muchir et al. 2000), congenital muscular dystrophy (MIM: 613205) (Quijano-Roy et al. 2008), dilated cardiomyopathy with conduction system defects (MIM: 115200) (Fatkin et al. 1999), autosomal recessive forms of axonal Charcot–Marie–Tooth (MIM: 605588) (De Sandre-Giovannoli et al. 2002), restrictive dermopathy (MIM: 275210) (Navarro et al. 2004), Dunnigan-type familial partial lipodystrophy (MIM: 151660) (Cao and Hegele 2000), mandibuloacral dysplasia (MIM: 248370) (Novelli et al. 2002), Hutchinson–Gilford progeria syndrome (HGPS) (MIM: 176670) (De Sandre-Giovannoli et al. 2003; Eriksson et al. 2003), atypical Werner syndrome (Chen et al. 2003), atypical HGPS (Hisama et al. 2011; Moulson et al. 2007), and cases displaying combination of abovementioned disorders (Alastalo et al. 2015; Saha et al. 2010).

In addition to the genes named above, biallelic pathogenic variants in *TOR1AIP1 (*MIM: 614512), encoding the integral nuclear membrane protein LAP1 (lamina-associated polypeptide 1), have been described in a family with three individuals affected by muscular dystrophy with variable cardiac involvement (Kayman-Kurekci et al. 2014), in a boy affected by dystonia, cerebellar atrophy, and cardiomyopathy (Dorboz et al. 2014), and in two siblings affected by cardiac failure and muscular dystrophy.(Ghaoui et al. 2016). Furthermore, a recent study identified a homozygous Palestinian founder variant, p.(Arg321*), in seven individuals from five likely related families affected by early onset multisystem anomalies with progeroid appearance and lethality in the 1st decade of life (Fichtman et al. 2019). Only those bearing the p.(Arg321*) had a loss of both LAP1B and LAP1C protein isoforms.

Here, we present two unrelated individuals with striking clinical similarities bearing biallelic loss-of-function variants in *TOR1AIP1* affecting both LAP1 isoforms. In comparison to the abovementioned study (Fichtman et al. 2019), one individual survived until 16 years of age and the other is alive at 34 years of age, respectively, and they were affected by multisystem anomalies and progeroid appearance that progressed with age. Moreover, we present the cellular characterization of primary fibroblasts of one of the affected individuals.

## Materials and methods

### Human subjects

All biological samples were obtained after written informed consent. The study was performed in accordance with the Declaration of Helsinki protocols and approved by the ethics committee of the respective institution.

### Cell lines

Primary human dermal fibroblast cultures were established from skin biopsies taken from the patient and three healthy donors (aged of 26, 34 and 35, respectively). Cells were cultured in Dulbecco’s modified Eagle’s medium (DMEM), supplemented with 10% fetal bovine serum (Life Technologies, 10270-106), 1% amphotericin B solution (Sigma, A2942) and 1% penicillin–streptomycin solution (Sigma, P4333) under 5% CO_2_ and at 37 °C. For all experiments, cells were trypsinized with trypsin–EDTA solution (Sigma, T3924) and seeded 24 h prior to the beginning of the experiment.

### Genetic analyses

DNA samples from whole blood were isolated by standard procedures. Exome sequencing of the proband 1 was performed using an Illumina GAIIx Sequencer with the Agilent SureSelect Human All Exon 50 Mb kit, as previously described (Lessel et al. 2014a). Approximately, 95% of target sequences covered at least tenfold and 89% 30-fold, with a mean coverage of 109×. Individual 2 was sequenced with the Agilent SureSelect Human All Exon V5 kit. The targeted regions were sequenced using the Illumina NextSeq 500 System with 150 bp paired-end reads. Following Broad’s Genome Analysis Toolkit (GATK) Best Practices guidelines, the DNA sequence was aligned and compared to the human genome build 19 (hg19/NCBI build 37). The average depth of coverage was 170x. The Cartagenia Bench Lab NGS software was used to filter and analyze sequence variants identified in the patient and compare them to the sequences of proband’s mother and sister. PCR and Sanger sequencing were performed as described previously (Lessel et al. 2015). All primer pairs are available upon request.

### Western blot analysis

Primary fibroblasts were lysed with protein lysis buffer containing 10 mM Tris–HCl, 150 mM NaCl, 2 mM EDTA, 1% Triton X-100, 10% glycerol, 10% proteinase inhibitors cocktail (Roche, 11 836 153 001) and 1 mM DTT. Total cell protein was obtained after 30 min of centrifugation (16,000 rpm). Protein concentration was determined with PierceTM BCA protein assay kit (Thermo Scientific, 23227) according to manufacturer’s instructions by reading absorbance at 562 nm with Epoch microplate spectrophotometer (BioTek). Protein volumes were adjusted to bring equal amounts on gel, mixed with BoltTM LDS sample buffer (Life Technologies, B0007) and BoltTM sample reducing agent (Life Technologies, B0009) according to manufacturer’s instructions. Afterwards, the samples were denaturated at 70 °C for 10 min and transferred to Bolt 4–12% Bis–Tris Plus Gel (Life Technologies, NW04120BOX). Samples were run at 165 V for 45 min in BoltTM MOPS SDS running buffer (Life Technologies, B0001). Proteins were transferred to the nitrocellulose membrane (Life Technologies, LC2000) at 10 V for 1 h and 15 min using Mini Blot Module (Life Technologies, B1000) and Bolt transfer buffer (Life technologies, BT0006) according to manufacturer’s instructions.

### Immunofluorescence

Primary fibroblasts were grown on glass coverslips, fixed with 4% formaldehyde, permeabilized with 0.1% Triton X-100, 0.1% Na citrate, blocked with 5% BSA in PBS and immunostained with the respective antibodies which were diluted in 5% BSA in PBS. Images were taken using Axioplan 2 imaging microscope (Zeiss, Jena, Germany) and captured using the AxioVision Imaging System (Zeiss, Jena, Germany). Nuclear abnormalities were estimated directly by visual observation at 100× magnification, in at least 100 cells per experiment.

### Antibodies

Primary antibodies used in this study were anti-beta actin (Abcam, ab8224), anti-ERK1/2 (Cell Signaling, 9102), anti-phospho ERK1/2 (Cell Signaling, 9101), anti-lamin A/C (Santa Cruz, sc-7292), anti-lamin B1 (Santa Cruz, sc-30264), anti-S6 kinase (Cell Signaling, 2317), anti-phospho S6 kinase (Cell Signaling, 2211) and anti-LAP1 (Atlas antibodies, HPA050546).

### Senescence-associated β-galactosidase activity assay

Cells were seeded for 24 h and the endogenous mammalian senescence-associated β-galactosidase activity (SAβ-gal) was evaluated using the Senescence β-Galactosidase Staining Kit (Cell Signaling), according to the manufacturer’s guidelines as previously described (Lessel et al. 2017b).

### Cell transfection with plasmid DNA

Plasmid-encoding Myc-Flag-tagged TOR1AIP1 (Origene, RC221686) was used in all experiments. 70.000 cells were seeded on 6-well cell culture plates for 24 h to ensure ~ 70% confluent cultures 24 h after seeding. 4 µg of plasmid DNA was transfected for additional 24 h using the Viromer Red reagent according to manufacturer’s instructions (Lipocalyx, Halle, Germany).

### RNA isolation and quantitative real-time PCR (qRT-PCR)

Total RNA was extracted with the RNAeasy mini kit (Qiagen) from primary fibroblasts that were in all in the same passage 8, and qRT-PCR with TaqMan probes was performed as previously described (Lessel et al. 2017a). TaqMan Gene Expression Assays (ThermoFisher Scientific) for the following human genes were used: *TOR1AIP1* (Assay ID: Hs01042342_m1), *LMNA* (Assay ID: Hs00153462_m1), *LMNB1* (Assay ID: Hs01059203_m1) and *GAPDH* (Assay ID: Hs02786624_g1).

### Statistical analysis

All analyses were performed using Prism 6 (GraphPad). Significance was determined with two-tailed Student’s *t* test. *P* values less than 0.05 were considered significant. Data represent mean ± SEM.

## Results

### Case reports

Individual 1 is a 34-year-old male who has since birth been regularly followed up at the University Medical Center Hamburg-Eppendorf. He is the first child of two, and his unaffected unrelated parents are of European descent. His 30-year-old brother is unaffected. Due to oligohydramnios, he was born at 34 weeks of gestation with a birth weight of 1940 g (− 0.95 SD), length of 40 cm (− 2.14 SD) and an occipitofrontal head circumference (OFC) of 31 cm (− 0.71 SD). Postnatal echocardiography revealed a ventricular septal defect (VSD) that spontaneously closed before his first birthday. In addition, hypospadias, undescended testes and congenital bilateral sensorineural hearing loss were diagnosed. Motor development was unaffected, with rolling over at 7 months and walking at 12 months. At the visit at the age of 8 years, we noted short stature and microcephaly [height 116.5 cm (− 2.43 SD) and OFC 48 cm (− 3.73 SD)]. We observed facial dysmorphisms that included long eyelashes, swinging eyebrows, telecanthus, epicanthus, anteverted nostrils, a long philtrum, and mandibular hypoplasia with dental crowding and irregular teeth. He had thin hair and developed bilateral cataracts and a mild hepatosplenomegaly. Further, a delay in speech development was noted, which was at that time regarded as a consequence of bilateral hearing loss. Extensive metabolic studies, bone age assessment and brain CT yielded normal results. EEG revealed a mild increase in cerebral excitability. Conventional chromosome analysis in lymphocytes gave normal results. Since his late 20s, he has had repeatedly elevated non-fasting glucose levels ranging from 127 to 201 mg/dl (reference 60–110 mg/dl), suggestive of type 2 diabetes; however, due to the lack of compliance, confirmatory tests have not been performed. In addition, he has had repeatedly elevated blood levels of non-fasting cholesterol ranging from 205 to 220 mg/dl (reference 150–200 mg/dl), LDH ranging from 320 to 480 U/l (reference 87–241 U/l), GGT ranging from 72 to 150 U/l (reference < 65 U/l), ALT ranging from 54 to 68 U/l (reference 10–50 U/l), and creatinine ranging from 1.64 to 3.60 mg/dl (reference 0.6–1.2 mg/dl). At the visit at the age of 31 years, he presented with bilateral hearing loss, mandibular hypoplasia with dental crowding and irregular teeth, scleroderma-like skin changes, Raynaud’s phenomenon, hand joint contractures, mild intellectual disability, muscular atrophy and proximal muscle weakness, dilated cardiomyopathy and chronic heart failure with significant reduction of left ventricular ejection fraction of 35% (normal range 50–70%) (Fig. S1). His weight was 40 kg and height 157 cm, resulting in a BMI of 16.23 kg/m^2^. The height of his mother, father and unaffected brother were 156 cm, 178 cm and 176 cm, respectively. His speech was slurred, although he had good speech understanding. Taken together, the combination of his symptoms led to the clinical diagnosis of a segmental progeroid syndrome. At the age of 32 years, following acute pneumonia, he experienced a fulminant cardiac crisis. In more detail, a cardiac decompensation with ventricular fibrillation occurred that required a short-term resuscitation including defibrillation. As a result, he developed acute renal failure with the need to start dialysis. An implantable cardioverter defibrillator (ICD) and an atrial (Demers) catheter for hemodialysis were implanted (Fig. S1b), followed by intensified anticongestive and antiarrhythmic therapy. Currently, at the age of 34 years, he still has severely reduced left ventricular function, receives dialysis for chronic renal failure, and experiences increasing difficulties in climbing stairs. Regarding diagnostic genetic analysis, chromosome analyses in blood lymphocytes did not demonstrate spontaneous chromosomal instability or hypersensitivity towards the genotoxic agent mitomycin C. Array CGH, and direct sequencing of *WRN*, *POLD1* and *LMNA* all gave normal results.

Individual 2 was a 16-year-old white male born after a full-term uncomplicated pregnancy to a 34-year-old G3P3 mother with birth weight of 3700 g. Both parents were unaffected and unrelated. His two siblings, age 17 and 15, respectively, were also healthy. A profound congenital sensorineural hearing loss was found at newborn screening. At 1 week of age, VSD was diagnosed, as well as an asymptomatic bicuspid pulmonary valve and persistence of a dilated pulmonary artery. The VSD was eventually closed. At 1.5 months of age, he developed bilateral cataracts and underwent surgical lens implants. He had G-tube insertion in early childhood due to feeding difficulty and failure to thrive. At his 4.5-year-old visit, he was noted to have microcephaly and short stature [OFC 47 cm (− 2.30 SD) and height 99 cm (− 1.74 SD)]. Other dysmorphic features included low-set and posteriorly rotated ears, deep-set eyes, thin nose, high-arched palate, long philtrum, thin upper lip, down-turned mouth, mandibular hypoplasia, and dental crowding. His development was globally delayed; he walked at 2.5 years, was nonverbal, but had learned 100–200 signs. There was no evidence of deterioration until he was around 11 years of age. At age 12, he developed bilateral ptosis, horizontal nystagmus, swallowing dysfunction, hypotonia, muscle weakness, gait changes, asymmetric and small thoracic size, mixed pectus carinatum and pectus excavatum, kyphoscoliosis, femoral anteversion, and cardiac arrhythmia. He had G-tube re-insertion due to difficulty swallowing. He also had surgery for bilateral ptosis and dental abnormalities. His symptoms continued to progress and at 15 years he was diagnosed with dilated cardiomyopathy with moderate left ventricular dysfunction. His left ventricular ejection fraction was 35.1% (normal range 50–70%) and frequent (2.2% of time) ventricular ectopic activity was detected through a 24-h Holter electrocardiogram (Fig. S2). At a clinical visit at age 16, he had chronic heart failure with ejection fraction only at 39%. He was mostly wheelchair-bound with global hypotonia and muscle weakness. Joint contractures were observed in his elbows, wrists, fingers and lower extremities. He also had nearly constant choreoathetoid movements and dystonic posture. His height was 149.8 cm (− 3.58 SD), weight 30.6 kg (− 5.17 SD) and head circumference 50 cm (− 4.27 SD). He died at age 16 years due to progressive heart failure. Quadriceps muscle biopsy at 1.5 years of age showed variable myofiber size with occasional small atrophic myofibers and centrally positioned nuclei; these findings were not considered specific for any specific disease processes. At the age of 12 years, brain MRI showed minimal periventricular signal abnormality but was otherwise within normal limits. CPK level was slightly decreased at 45 (normal: 55–215) and lactic acid was slight elevated at 22.6 (normal: 4.5–18). Extensive metabolic and biochemical workup was unrevealing, including plasma amino acids, acylcarnitine, carnitine, GALT, urine amino acids, organic acids, MPS type 1, GAG, lysosomal enzyme, carbohydrate deficient transferrin, N-glycan, O-glycan, 7-dehydrocholesterol, and very-long-chain fatty acids. Negative genetic testing included FISH, karyotype, mitochondrial DNA genome sequence, cytogenomic microarray, and direct sequencing of *GJB2*, *GJB6*, *SLC52A3*, *SLC52A2*, and *SLC52A1*.

**Fig. 1:**
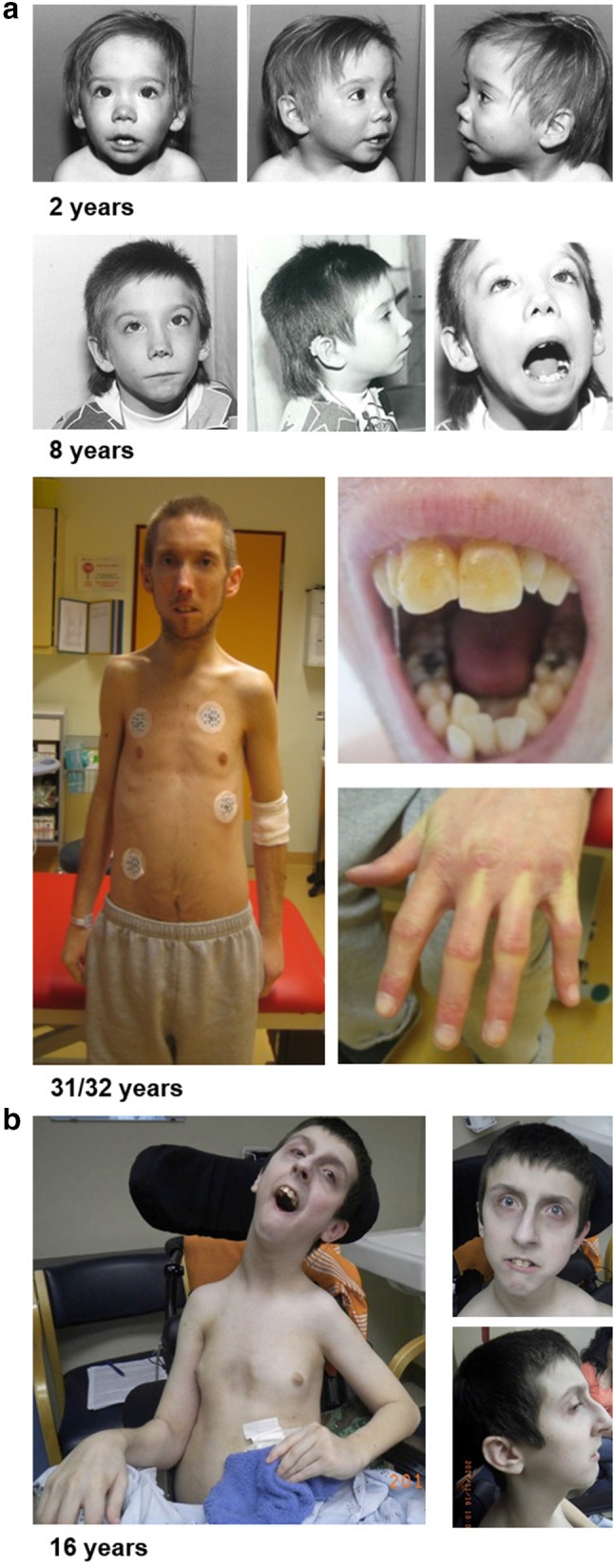
Facial and body images of the here presented individuals. Images of individual 1 at the ages of 2 years, 8 years and 31/32 years. Note the small chin and dental crowding indicative for mandibular hypoplasia (**a**). Images of individual 2 at 16 years (**b**)

Taken together, both individuals presented with congenital bilateral hearing loss and ventricular septal defect, followed by development of bilateral cataracts, mild to moderate developmental delay, microcephaly, mandibular hypoplasia with dental crowding and irregular teeth, relative short stature, progressive muscular atrophy, joint contractures, dilated cardiomyopathy, and reduced left ventricular ejection fraction followed by severe chronic heart failure (Fig. 1, Table 1, Fig. S1 and S2). Thus, both prematurely developed a combination of symptoms commonly observed in the elderly, i.e., bilateral cataracts, progressive muscular atrophy and early onset cardiomyopathy, similar to the findings in diverse segmental progeroid syndromes (Lessel and Kubisch 2019; Martin 1978, 2005).

**Table 1:** Clinical characteristics of individuals with *TOR1AIP1* variants

Reference	Individual 1 of this article	Individual 2 of this article	Kayman-Kurekci et al.	Dorboz et al.	Ghaoui et al.	Fichtman et al.
Number of subjects	1	1	3	1	2	7
Congenital heart defect	+	+	–	–	–	6/7
Hearing loss	Congenital	Congenital	–	–	–	7/7, congenital 3/7
Mandibular hypoplasia	+	+	*	–	–	0/7
Bilateral cataracts	+	+	–	–	–	7/7
Developmental delay	Mild	Moderate	–	–	1/2	Severe 7/7
Short stature	+	+	*	–	–	7/7
Microcephaly	+	+	–	–	–	7/7
Joint contractures	+	+	3/3	–	–	0/7
Muscle weakness	+	+	3/3	–	2/2	7/7
Dilated cardiomyopathy	+	+	1/2	+	2/2	?
Chronic heart failure	+	+	–	+	2/2	?
Skin manifestations	Scleroderma-like skin changes	–	–	–	–	Hypertrichosis 2/7
Raynaud’s phenomenon	+	–	–	–	–	0/7
Feeding difficulties	+	+	?	?	?	7/7
Gastrointestinal anomalies	–	–	?	?	?	5/7
Renal anomalies	+	–	?	?	?	2/7
Dystonia	–	+−	–	+	–	0/7
Brain MRI/CT	Normal results	Minimal periventricular signal abnormality	-?	Cerebellar atrophy	Normal results (1/2) ? (1/2)	Global parenchymal brain atrophy 4/4
*TOR1AIP1* mutations	c.945_948delCAGT/c.1331G > C p.(Gln315Hisfs*9)/ p.(Arg444Pro)	c.649G > T/c.724delG p.(Glu217*)/ p.(Asp242Ilefs*17)	c.186delG/c.186delG p.Glu62*fs*Ter*25/ p.Glu62*fs*Ter*25	c.1448A > T/c.1448A > T p.Glu482Ala/p.Glu482Ala	c.127delC/c.1181T > C p.Pro43fs*15/p.Leu394Pro	c.961C > T/ c.961C > T p.Arg321*/ p.Arg321*

+, Present; −, absent; *not reported in the manuscript, however, observed to be presented in Fig. 1b (Kayman-Kurekci et al. 2014); ?, not reported

### Genetic analyses

**Fig. 2:**
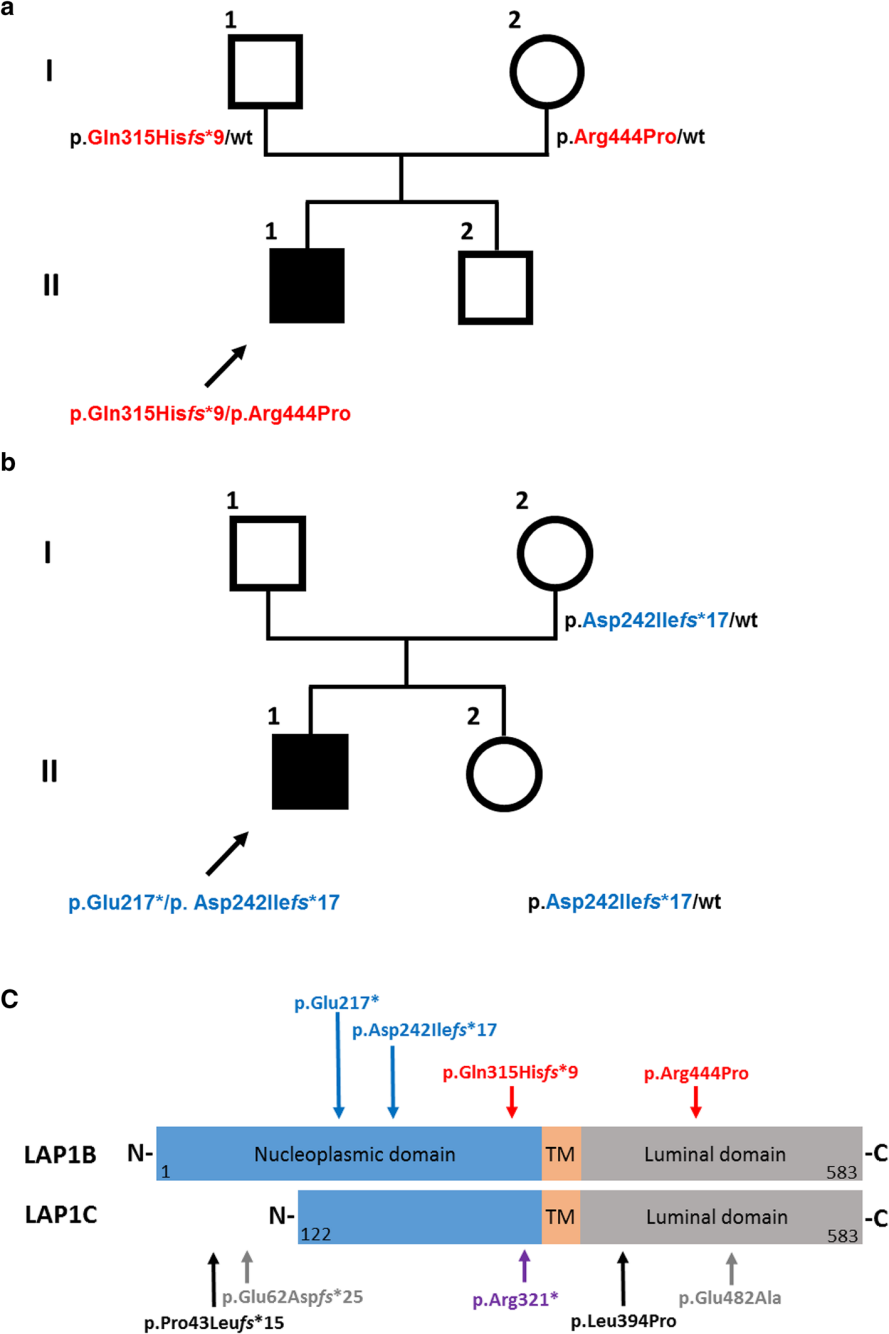
Genetic analysis of both families. Pedigree of families 1 and 2, respectively. Filled and open symbols denote affected and healthy individuals, respectively; arrow further indicates affected individuals. *TOR1AIP1* genotypes are shown next to each symbol (**a**, **b**). Schematic protein structure of both major LAP1 isoforms, LAP1B and LAP1C, respectively. The position of the variants identified in this study is shown above the protein structure. Variants identified in individual 1 are marked with red vertical arrows and variants identified in individual 2 are marked with blue arrows. All variants affect both LAP1 isoforms. Previously identified *TOR1AIP1* pathogenic variants in independent affected individuals are marked beneath the protein structure with black and gray arrows, denoting compound heterozygous and homozygous variants, respectively. Note that p.Pro43Leufs*15 and p.Glu62Aspfs*25 affect only the LAP1B isoform. The homozygous nonsense variant p.Arg321* (violet arrow) affects both isoforms. (TM, transmembrane domain; N-terminus; C-terminus) (**c**)

To identify the putative genetic cause, whole-exome sequencing (WES) was performed with DNA samples of both affected individuals. Bioinformatic filtering of WES data in individual 1 for putatively de novo variants detected 42 heterozygous alterations that have previously not been annotated in dbSNP, the ExAC database or the 1000 Genomes data; however, subsequent Sanger sequencing with DNA samples of both healthy parents and individual 1 revealed that each of these variants was inherited (Table S1). Further, we did not detect rare candidate variants (minor allele frequency MAF < 0.001) following an X-linked recessive mode of inheritance or any putatively pathogenic homozygous alterations. However, we identified two genes with rare (minor allele frequency MAF < 0.001) double heterozygous variants affecting *GMPPA* and *TOR1AIP1* (Table S2). Segregation analysis revealed that both heterozygous variants affecting *GMPPA* were inherited from the unaffected father, thus excluding a causative role. On the contrary, we confirmed a paternally inherited c.945_948delCAGT, p.(Gln315Hisfs*9), and a maternally inherited c.1331G > C, p.(Arg444Pro), mutation in *TOR1AIP1* (NM_001267578.1) (Fig. 2a). The frameshift variant is predicted to lead to a premature termination codon, likely activating nonsense-mediated mRNA decay. The arginine residue at position 444 is highly conserved in LAP1 orthologues; accordingly, p.(Arg444Pro) is classified as “probably damaging” with a maximum score of 1.0 by Polyphen-2. It also has a very high phred-scaled CADD (v1.3) score of 29.6, consistent with a pathogenic variant.

Bioinformatic filtering of WES data in individual 2 also revealed two likely pathogenic variants in *TOR1AIP1*, namely, a maternally inherited variant c.724delG, p.(Asp242Ilefs*17) and a second heterozygous variant c.649G > T, p.(Glu217*). As paternal DNA was not available for sequencing, we could only infer that this second variant is likely to have been inherited from his father, as it is absent in both his mother and unaffected sister. His unaffected sister is a carrier of the c.724delG, p.(Asp242Ilefs*17) (Fig. 2b). Similar to the p.(Gln315Hisfs*9), these frameshift and nonsense variants are predicted to lead to a premature termination codon, likely activating nonsense-mediated mRNA decay as was recently shown for the homozygous p.(Arg321*). According to the gnomAD database, all four identified variants are exceedingly rare: p.(Glu217*) was observed 3 times, p.(Asp242Ilefs*17) was observed 1 time, p.(Arg444Pro) was observed 16 times, whereas p.(Gln315Hisfs*9) was not annotated in this database. Taken together, the genetic data provided strong support for the causality of these *TOR1AIP1* variants.

### Low protein levels of LAP1B/LAP1C accompanied by low protein and mRNA levels of lamin A/C and lamin B1 in dermal fibroblasts of individual 1

**Fig. 3:**
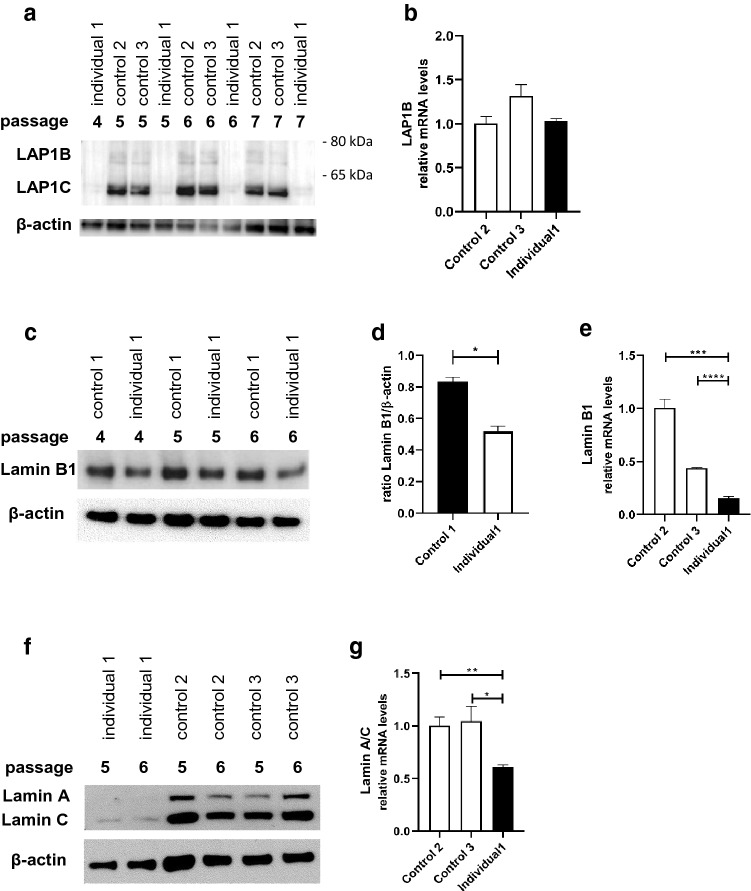
Reduced expression of LAP1B, LAP1C, lamins B1, A and C in fibroblasts of individual 1. Protein analysis in four subsequent fibroblast passages (P4-7) of individual 1 and three subsequent fibroblast passages (P5-7) of two control samples. Analyses of LAP1B and LAP1C, normalized versus β-actin in individual 1’s dermal fibroblasts and a control sample (control 1). Note the absence of both LAP1 isoforms in individual 1’s dermal fibroblasts (**a**). qRT-PCR analysis of *TOR1AIP1* in individual 1, and control 2 and 3. The mRNA values were normalized versus *GAPDH* (**b**). Protein analysis in three subsequent fibroblast passages (P4-6) of lamin B1 normalized versus β-actin in individual 1´s dermal fibroblasts and a control sample (control 1). Note the low lamin B1 levels relative to control sample, in all three passages (**c**). Quantification of experiments shown in (**c**). Data are presented as mean ± SEM. Two-tailed Student’s *t* test (**d**). qRT-PCR analysis of *LMNB1* in individual 1 and control 2 and 3. The mRNA values were normalized versus GAPDH (**e**). Expression analyses of lamin A and C, in two subsequent fibroblast passages (P5-6) in individual 1’s dermal fibroblasts and two further control samples (controls 2 and 3). Note the low lamin A and C levels relative to control samples, in both passages (**f**). qRT-PCR analysis of *LMNB1* in individual 1, and control 2 and 3. The mRNA values were normalized versus GAPDH (**g**)

To corroborate our genetic findings and provide further evidence for the causality of the *TOR1AIP1* variants identified in individual 1, we established primary dermal fibroblasts of individual 1. We additionally established primary dermal fibroblasts of three unaffected individuals of similar age (controls 1–3, aged 26, 34 and 35, respectively). Individual 2 unfortunately deceased prior to skin biopsy, thus we were unable to analyze his primary cell lines. It is, however, worth noting that both of the variants identified in individual 2 lead to premature stop codons which are more N-terminal than the p.(Arg321*), for which a nonsense-mediated mRNA decay was recently shown (Fichtman et al. 2019). Given that the individuals bearing the homozygous p.(Arg321*) show a complete loss of both LAP1 protein isoforms, LAP1B and LAP1C, by immunoblotting, we first analyzed these isoforms in individual 1. Surprisingly, given that individual 1 survived far longer and that he bears a missense variant in addition to a frameshift one, we were not able to detect either LAP1B or LAP1C in any of the four subsequent passages (passages 4–7). In contrast, both LAP1B and LAP1C were detected in control fibroblasts (Fig. 3a, Fig. S3). Quantitative real-time PCR (qRT-PCR) did not reveal a difference in the mRNA level between individual 1 and two controls (Fig. 3b). However, structure- and sequence-based predictors of the effect of single point variations on protein stability I-Mutant v2.0 (Capriotti et al. 2005), iPTREE-STAB (Huang et al. 2007), Site Directed Mutator (Pandurangan et al. 2017) and Cupsat (Parthiban et al. 2006) all predicted a large decrease in stability, suggesting that the p.Arg444Pro results in an unstable protein.

We next analyzed protein levels of two proteins within the nuclear lamina which support the inner nuclear membrane, lamin B1 and lamin A/C, in three subsequent passages (passages 4–6) of individual 1 fibroblasts and fibroblasts from an unaffected control individual. We observed, relative to fibroblasts from the healthy control individual, lower levels of lamin B1 (Fig. 3c, d, Fig. S4) and very low basal levels of both lamins A and C, even after prolonged exposure (Fig. S5). Given that previous studies did not reveal reduced lamin A/C levels (Fichtman et al. 2019; Ghaoui et al. 2016), we additionally analyzed lamin A and C levels in primary fibroblasts of two further independent unaffected individuals and observed again much lower levels of lamins A and C (Fig. 3f). Similarly, qRT-PCR analyses revealed significant reduction of *LMNA* and *LMNB1* in the mRNA level (Fig. 3e, g). Thus, our data provide further evidence for LAP1 involvement in the regulation of lamins, exemplifying the complexity of the interconnected nuclear membrane system.

### Nuclear abnormalities in patient’s dermal fibroblasts

**Fig. 4:**
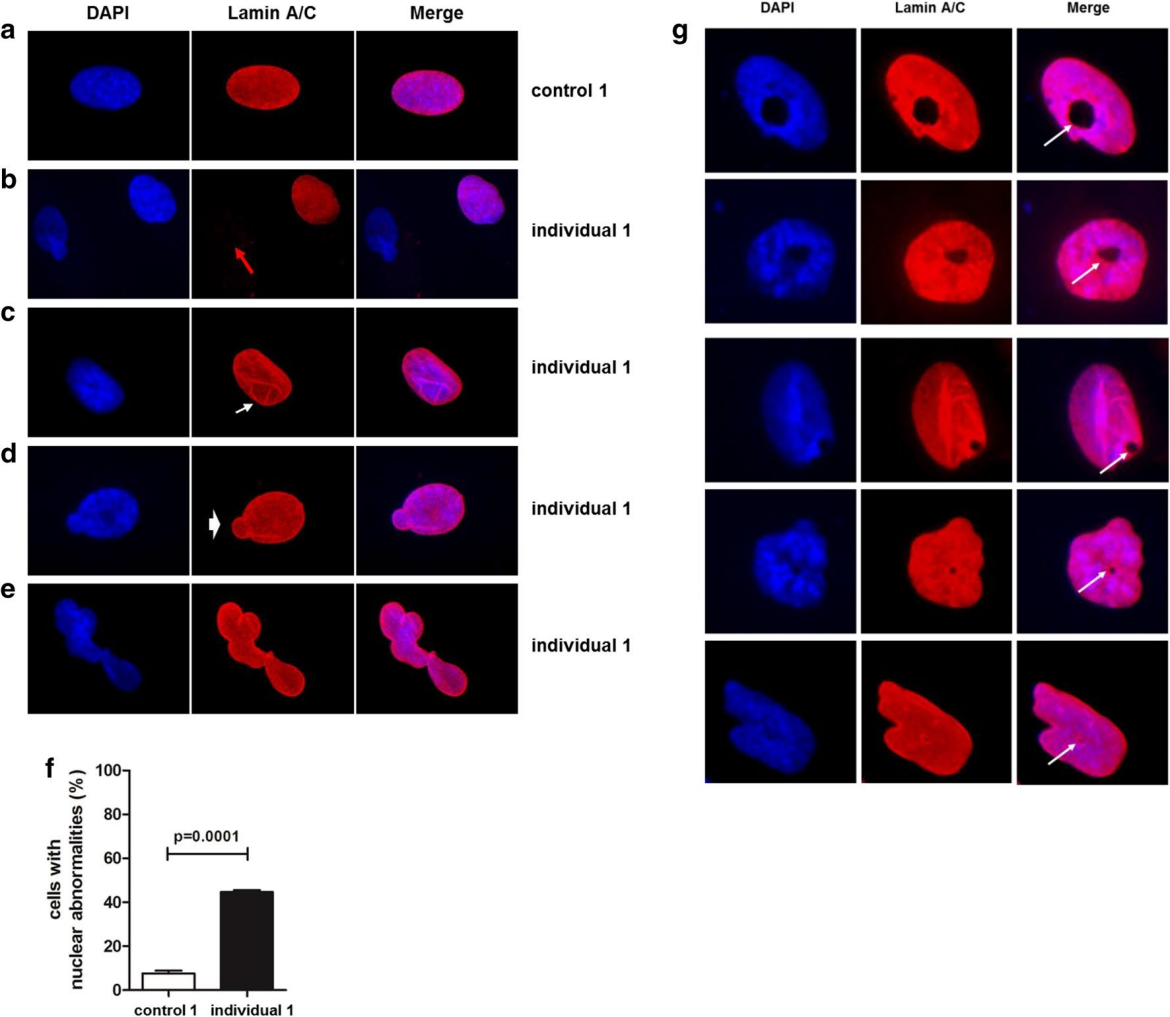
Nuclear abnormalities and uneven distribution of lamin A/C in fibroblasts from individual 1. Conventional immunofluorescence microscopy with DAPI (blue) and anti-lamin A/C antibody (red). Images were taken using 100× magnification. Representative images of control fibroblasts (**a**), of patient’s fibroblasts with complete absence of lamin A/C staining (red arrow) (**b**), of patient’s fibroblasts showing invagination of the nuclear envelope (white arrow) (**c**), of patient’s fibroblasts showing a nuclear bleb (white arrowhead) (**d**), of patient’s fibroblasts showing a complex nuclear lobulation abnormality (**e**) are shown. Quantification of various nuclear abnormalities in individual 1’s dermal fibroblasts (representative images are shown in Fig. 5). Bar graphs is representative of three independent experiments, around 100 cells were analyzed per experiment. *p* < 0.0001 (control vs. patient). Data are presented as mean ± SEM. Two-tailed Student’s *t* test (**f**). Representative images of nuclear cytoplasmic channels. Channels are marked with white arrows (**g**)

Given the role of LAP1B in the maintenance of nuclear membrane morphology (Kim et al. 2010; Serrano et al. 2016) and previous findings in individuals affected by a *TOR1AIP1*-associated disorder (Fichtman et al. 2019; Kayman-Kurekci et al. 2014), we performed immunofluorescence analysis of fibroblasts derived from individual 1 and an unaffected control individual. In control fibroblasts, all cells displayed positive lamin A/C staining, and the majority, around 90%, of nuclei were oval or ovoid (Fig. 4a). On the contrary and in line with reduced lamin A/C protein levels, in fibroblasts of individual 1, we observed loss of lamin A/C staining in roughly 18% of analyzed nuclei (Fig. 4b), which was not observed in control fibroblasts. These findings are comparable to the observed effect in primary fibroblasts of individuals bearing the homozygous p.Arg321* variant that also displayed reduced lamin A/C staining (Fichtman et al. 2019). Further, we observed invagination of the nuclear envelope (Fig. 4c), likely due to lamin A/C aggregates both at the nuclear periphery and in the nucleoplasmic area, nuclear blebs (Fig. 4d) and complex nuclear lobulations (Fig. 4e), likely due to twisting of the nuclear envelope, in around 44% of analyzed nuclei of individual 1 (Fig. 4f). With ~ 90% the gross majority of these abnormalities consisted of nuclear invaginations. These nuclear abnormalities largely resemble the findings in other progeroid forms of nuclear envelopathies (Carrero et al. 2016; Cau et al. 2014) and are in accordance with the findings in LAP1B-deficient mice (Kim et al. 2010). Additionally, comparable to the previous findings we observed in around 4% of individual 1’s primary fibroblasts nuclear holes that differed in shape and dimensions (Fig. 4g), which according to the previous data likely resemble cytoplasmic channels traversing the nucleus (Fichtman et al. 2019). Such nuclear channels were not observed in control cell lines, and we have not observed them before either in primary cell lines of unaffected or in individuals affected by various progeroid features (Hisama et al. 2011; Lessel et al. 2014b, 2017b; Marbach et al. 2019).

### Premature senescence in patient’s dermal fibroblasts

**Fig. 5:**
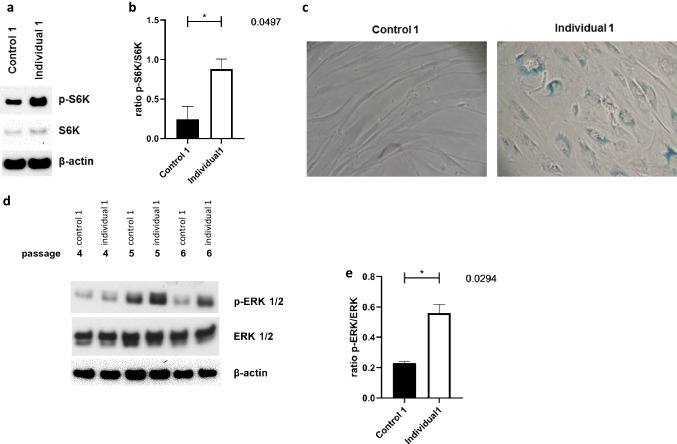
Senescence phenotype and abnormal activation of extracellular signal-regulated kinase (ERK1/2) in fibroblasts from individual 1. Protein analyses of phosphorylated S6 kinase (p-S6k) and total S6 kinase (S6k) normalized versus β-actin. Note the increased levels of p-S6k in patient’s fibroblasts relative to control sample in passage 6 (**a**). Quantification of phosphorylated S6 kinase (p-S6k) versus total S6 kinase (S6k) in three independent experiments in three subsequent fibroblast passages (P4-6), as shown in (**a**). Data are presented as mean ± SEM. Two-tailed Student’s *t* test (**b**). Fibroblasts shown were in passage 7. Representative image of increased SA-β-galactosidase staining in individual 1’s fibroblasts relative to control sample. Fibroblasts shown were in passage 7. Image was taken using 20× magnification (**c**). Protein analysis in three subsequent fibroblast passages (P4-6). Analyses of phosphorylated ERK1/2 (p-ERK1/2) and total ERK1/2 normalized versus β actin in individual 1’s dermal fibroblasts and a control sample. Note the increased levels of p-ERK1/2 in patient’s fibroblasts relative to control sample (**d**). Quantification of experiments in three subsequent fibroblast passages (P4-6), as shown in (**d**). Data are presented as mean ± SEM. Two-tailed Student’s *t* test (**e**)

Since lamin B1, besides being an integral part of the nuclear envelope, is a well-established marker of cellular senescence (Shimi et al. 2011) we asked whether this might point to premature senescence in fibroblasts of individual 1. We therefore analyzed by immunoblotting, the mTOR (mammalian target of rapamycin) pathway, investigating both basal and phosphorylated S6, a ribosomal protein that is phosphorylated in response to mTOR activation, in passage 7. We observed significantly elevated levels of phosphorylated S6 kinase (pS6k) in fibroblasts of individual 1 (Fig. 5a, b, Fig. S6) providing further evidence for premature senescence of these fibroblasts (Blagosklonny 2012). In addition, using the senescence-associated beta-galactosidase (SA-βgal), we confirmed the senescent state of the primary cell lines of individual 1 (Fig. 5c). This cellular phenotype rapidly advanced and his fibroblasts entered replicative senescence, e.g., stopped dividing, at passage 10. In stark contrast and as expected, control cells—managed in the same manner as individual 1’s fibroblasts—proliferated beyond passage 40. Senescence is not only a common finding in several segmental progeroid syndromes (Carrero et al. 2016; Cau et al. 2014; Eriksson et al. 2003; Salk et al. 1981; Tivey et al. 2013), but has additionally been established as one of the main hallmarks of “physiological” aging (Lopez-Otin et al. 2013). Therefore, these data further suggest the clinical diagnosis of a segmental progeroid features on the cellular level.

### Abnormal activation of extracellular signal-regulated kinase (ERK1/2) in patient’s dermal fibroblasts

A common theme that has emerged amongst nuclear envelopathies is the deregulation of the MAPK signaling pathway, and more specifically the marked activation of the ERK1/2 pathway, in several in vitro models (Muchir et al. 2007a, 2009; Tapia et al. 2015; Zhou et al. 2017). The MAPK signaling pathway is activated in Emery–Dreifuss muscular dystrophy mice models, bearing either the Lmna H222P mutation (Muchir et al. 2007b) or an EMD null allel (Muchir et al. 2007a). Moreover, in vitro down-regulation of lamin A, emerin (Muchir et al. 2009) or LEMD2 (Huber et al. 2009) resulted in activation of extracellular signal-regulated kinase (ERK1/2), similar to recent findings in *SYNE1*-associated diseases (Zhou et al. 2017). Activation of the MAPK signaling pathway was also observed in Lemd2-deficient mice (Tapia et al. 2015). These findings prompted us to analyze basal ERK1/2 and phosphorylated ERK1/2 levels in primary cells of individual 1 by immunoblotting. We found that in all three subsequent passages the level of phosphorylated ERK1/2 was significantly higher in individual 1’s fibroblasts as compared to fibroblasts of a healthy control (Fig. 5d, e, Fig. S7). These results further support the role of nuclear envelope proteins in the regulation of cell signaling.

### Ectopic expression of wild-type TOR1AIP1 mitigates the observed cellular phenotypes

**Fig. 6:**
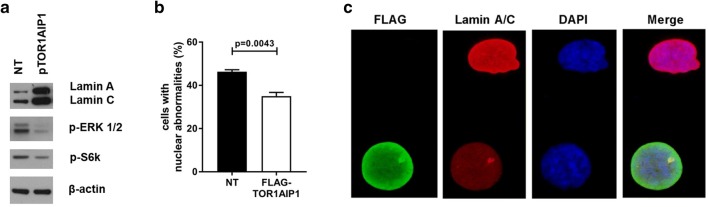
Ectopic expression of wild-type TOR1AIP1 mitigates the observed cellular phenotypes in fibroblasts from Individual 1. Representative western blot images of lamin A and C, p-ERK1/2 and p-S6k, normalized versus β-actin in patient’s dermal fibroblasts untreated or transfected with 4 µg of wild-type Myc-Flag-tagged TOR1AIP1 plasmid (**a**). Quantification of various nuclear abnormalities in non-transfected (patient NT) and with 4 µg of wild-type Myc-Flag-tagged TOR1AIP1 plasmid (patient + FLAG-TOR1AIP1) patient’s dermal fibroblasts. Bar graphs is representative of 3 independent experiments, around 100 cells were analyzed per experiment. *p *< 0.0043 (control vs. patient). Data are presented as mean ± SEM. Two-tailed Student’s *t* test (**b**). Representative conventional immunofluorescence microscopy image with anti-FLAG antibody (green), anti-lamin A/C antibody (red) and DAPI (blue) of an untransfected cell bearing a nuclear bleb, and a cell transfected with 4 µg of wild-type Myc-Flag-tagged TOR1AIP1 plasmid. Image was taken using 100× magnification (**c**)

As described above, cellular characterization of primary fibroblasts derived from individual 1 revealed absent LAP1B and LAP1C, constitutively low lamin A/C levels, aberrant nuclear morphology, premature cellular and replicative senescence, and abnormal ERK1/2 activation. While neither premature senescence nor ERK1/2 levels had been analyzed in previously reported human *TOR1AIP1* pathologies, one study reported mildly elevated lamin A/C levels in both skeletal and cardiac muscles (Ghaoui et al. 2016). Individuals bearing the homozygous p.(Arg321*) did not display altered lamin A/C levels (Fichtman et al. 2019). In addition, the nuclear abnormalities, although in accordance with the findings in LAP1-deficient mice (Kim et al. 2010) and being reported in human *TOR1AIP1* pathologies before (Fichtman et al. 2019; Kayman-Kurekci et al. 2014), have not been observed in all individuals (Dorboz et al. 2014). To further confirm that the cellular phenotypes observed in individual 1 are caused by the *TOR1AIP1* mutations and thereby to confirm their causality, we transiently transfected his fibroblasts with a plasmid containing the wild-type *TOR1AIP1* open reading frame (ORF). Using polyethylenimine polyplex transfection technology (Viromer RED), we achieved a transfection efficiency of around 20% in passage 8 of individual 1’s fibroblasts, as revealed by immunofluorescence analysis with anti-FLAG (Fig. S8). Immunoblotting analyses revealed that ectopic expression of TOR1AIP1 resulted in robust stabilization of lamin A/C levels as well as a partial reduction in both pS6k, as a marker of premature senescence, and pERK1/2 levels (Fig. 6a). In addition, it resulted in a significantly reduced rate of aberrant nuclei in individual 1´s fibroblasts as compared to non-transfected cells (*P* = 0.0043), and we observed no nuclei lacking lamin A/C staining (Fig. 6b, c). These findings strongly suggest the causal role of the *TOR1AIP1* mutations identified in individual 1.

## Discussion

Segmental progeroid syndromes, defined as syndromes with signs of premature aging affecting more than one tissue or organ (Martin 1978), are highly heterogenous genetic disorders. Studies utilizing next-generation sequencing (NGS) approaches have revealed several novel progeroid genes in recent years (Ehmke et al. 2017; Jay et al. 2016; Lessel et al. 2014b, 2017b, 2018; Marbach et al. 2019; Paolacci et al. 2018; Puente et al. 2011; Schrauwen et al. 2015; Wambach et al. 2018), and broadened the spectrum of phenotypes of many known progeroid conditions and the number of associated genetic causes (Lessel et al. 2014a, 2015; Rodriguez-Garcia et al. 2018; Soria-Valles et al. 2016). Here we describe two individuals affected by multisystem anomalies and progeroid features that progressed with age, bearing compound heterozygous pathogenic variants in *TOR1AIP1* affecting both LAP1 isoforms. Recent studies have associated biallelic *TOR1AIP1* mutations with several conditions somewhat mimicking the clinical heterogeneity and phenotypic continuum of *LMNA*-associated disorders (Bertrand et al. 2011). Both individuals presented herein share some clinical features with the individuals affected by isolated LAP1B deficiency, i.e., a severe form of cardiomyopathy and muscular atrophy. The fulminant cardiac crisis, accompanied by kidney failure in individual 1 and death in individual 2, further highlights the importance of tight cardiac surveillance for *TOR1AIP1* mutated individuals (Ghaoui et al. 2016). On the other hand, early onset multisystem anomalies, such as congenital hearing loss, bilateral cataracts, developmental delay, growth retardation and progressive progeroid appearance have been documented only in individuals bearing the homozygous p.(Arg321*), who have a combined LAP1B and LAP1C deficiency (Fichtman et al. 2019). Mandibular hypoplasia with dental crowding and irregular teeth, scleroderma-like skin changes, Raynaud’s phenomenon, most of which are commonly observed in other segmental progeroid syndromes (Cau et al. 2014; Lessel et al. 2015), have not been previously associated with *TOR1AIP1* before (Table 1). It is worth noting that we cannot exclude the possibility that some of these features might have been present in previously reported individuals but were not documented. For instance, the image of the index patient reported by Kayman-Kurekci et al. shows a young woman with somewhat small mandible and gracile habitus that is not further mentioned in their manuscript (Kayman-Kurekci et al. 2014). Our findings further suggest that a combined LAP1B and LAP1C deficiency results in early onset, multisystem anomalies as compared to isolated LAP1B deficiency. However, we cannot completely exclude the possibility that any of the present or previously reported individuals (Fichtman et al. 2019) with combined LAP1B and LAP1C deficiency bears additional variant or epigenetic change in other genes that may modify the phenotype.

To gain further evidence that the *TOR1AIP1* variants in individual 1 are causative, we performed functional characterization of patient-derived dermal fibroblasts. The paternal variant creates a frameshift l, whereas the maternal missense variant affects a highly conserved arginine residue. Immunoblotting revealed strongly reduced LAP1B and LAP1C transcript levels arguing that both inherited variants result in at least a strong hypomorphic allele. Moreover, we observed constitutively low lamin A/C levels in several fibroblast passages accompanied by complete loss of lamin A/C immunofluorescence staining in around 18% of the patient cells. Although LAP1 is a known interactor of lamin A/C (Kubben et al. 2010), this is in contrast to previously published data where lamin A/C levels were mildly elevated in both skeletal and cardiac muscles of a single patient (Ghaoui et al. 2016) or not altered (Fichtman et al. 2019). However, the latter study did identify reduced lamin A/C staining in individuals bearing the homozygous nonsense alteration (Fichtman et al. 2019). Furthermore, similar to the previous findings (Fichtman et al. 2019) we also observed nuclear cytoplasmic channels in primary fibroblasts of the individual 1. Further characterization of the primary fibroblasts of the individual 1 revealed that these cells prematurely enter senescence, as revealed by deregulation of LMNB1 and phosphorylated S6 kinase, positive SA-βgal staining and early replicative senescence. To confirm that the cellular phenotypes observed in our study are indeed due to the *TOR1AIP1* variants, we have over-expressed wild-type TOR1AIP1 in fibroblasts and observed a partial to complete rescue of several cellular phenotypes, thus providing strong evidence for their causality.

In conclusion, using a combination of exome sequencing and segregation analyses followed by functional characterization of primary fibroblasts of individual 1, we identified biallelic pathogenic variants in *TOR1AIP1* resulting in combined LAP1B and LAP1C deficiency as the genetic cause of two individuals affected by multisystem anomalies and progressive progeroid features. Thus, we further expand the clinical phenotype underlying *TOR1AIP1*-associated disorders and suggest that individuals bearing combined LAP1B and LAP1C deficiency can survive beyond the 1st decade of life.

## Electronic supplementary material

Below is the link to the electronic supplementary material.

Supplementary material 1 (DOCX 2970 kb)

## Data Availability

The identified *TOR1AIP1* variants have been deposited to the Leiden Open (source) Variation Database (LOVD) (http://www.lovd.nl/3.0/home) and will be available upon manuscript acceptance. The raw whole-exome sequencing data that support the findings in affected individual cannot be made publicly available for reasons of affected individual’s confidentiality. Qualified researchers may apply for access to these data, pending institutional review board approval. All other data generated or analyzed during this study are included in this published article (and its supplemental data).
