# Supplementary material for: Two novel cases further expand the phenotype of TOR1AIP1-associated nuclear envelopathies
Source: Hum Genet. 2020 Feb 13;139(4):483–98. doi: 10.1007/s00439-019-02105-6 (PMC7078146; doi:10.1007/s00439-019-02105-6)
Supplement: Supplementary file 1 — Supplementary material 1 (DOCX 2970 kb) [file 439_2019_2105_MOESM1_ESM.docx]

**Supplementary material for:**

**Two novel cases further expand the phenotype of *TOR1AIP1-*associated nuclear envelopathies**

Ivana Lessel^1^, Mei-Jan Chen^2^, Sabine Lüttgen^1^, Florian Arndt^3^, Sigrid Fuchs^1^, Stefanie Meien^1^, Holger Thiele^4^, Julie R. Jones^5^, Brandon R. Shaw^2^, David K. Crossman^2^, Peter Nürnberg^4,6,7^, Bruce R. Korf^2^, Christian Kubisch^1^, Davor Lessel^1#^

#Correspondence should be addressed to DL ([d.lessel@uke.de](mailto:d.lessel@uke.de))

**Figure S1. Cardiac phenotype of individual 1.**


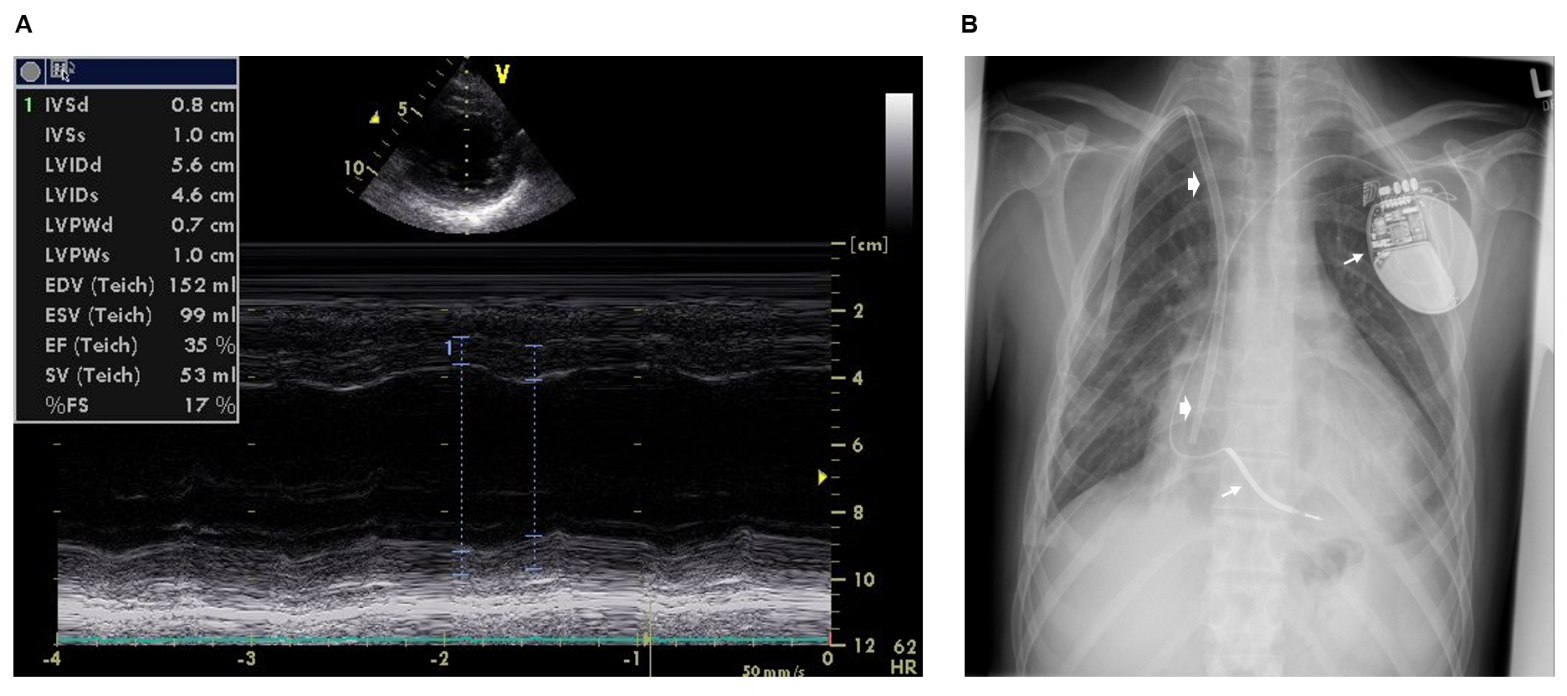


The image of echocardiography of individual 1 shows a significant reduction of the left ventricular function with a fractional shortening (FS) of 17% (standard values would be over 27%) and reduction of the left ventricular ejection fraction (EF) of 35% (according to the guidelines of the American Heart Association values under 40% are regarded as reduced). Left ventricle end-diastolic diameter (LVEDD, in the figure depicted as LVIDd) is enlarged with 5.6 cm (range 4,37 – 5.5 3cm; z-score: 2.18) (**a**). The chest x-ray shows the cardiomegaly (cardiothoracic ratio (CTR) 0.7 (normal measurement less than 0.5). In addition, the implanted cardioverter defibrillator (white arrows) with the correct position of the ICD lead is shown in the right ventricle, and the inserted dialysis catheter (white arrowheads) is seen in the right atrium (**b**).

**Figure S2. Cardiac phenotype of individual 2.**


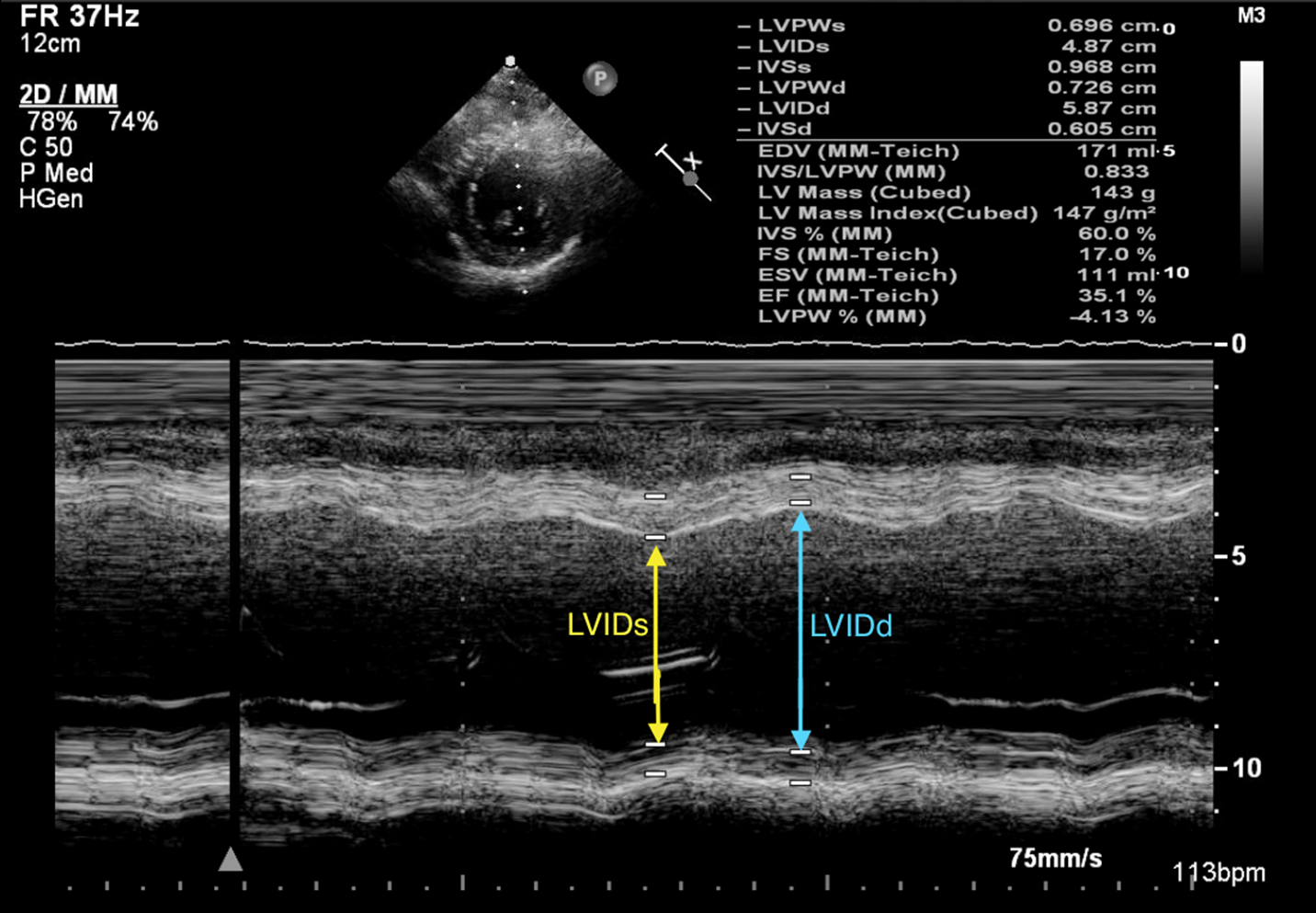


The image of echocardiography of individual 2 shows a significant reduction of the left ventricular function with a fractional shortening (FS) of 17% (standard values would be over 27%) and reduction of the left ventricular ejection fraction (EF) of 35% (according to the guidelines of the American Heart Association values under 40% are regarded as reduced). Left ventricle end-diastolic diameter (LVEDD, in the figure depicted as LVIDd) is enlarged with 5.87 cm (range 3.91 – 5.01 cm; z-score: 4.74).

**Fig. S3. *TOR1AIP1* variants in individual 1 result in the loss of both LAP1B and LAP1C isoforms (full blot corresponding to Fig. 3a)**


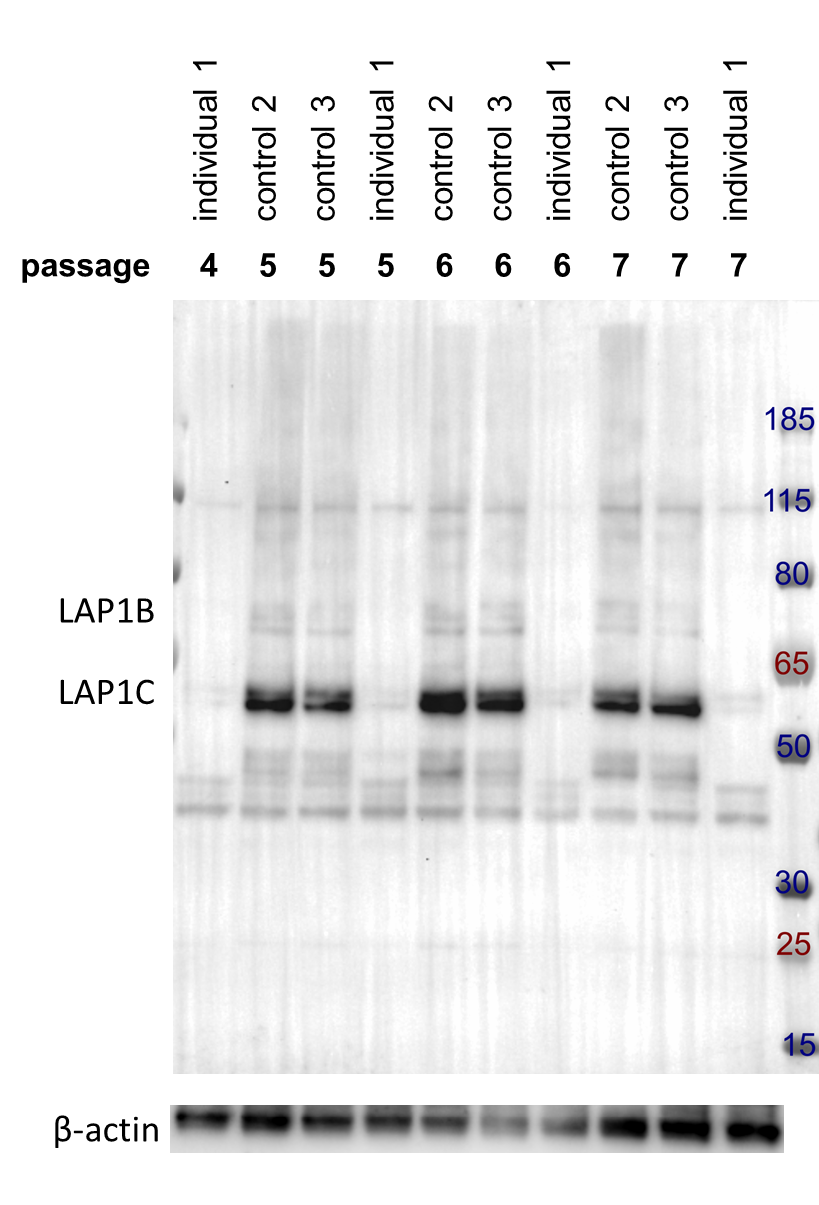


Protein analysis in four subsequent fibroblast passages (P4-7) of individual 1 and three subsequent fibroblast passages (P5-7) of two control samples. Analyses of LAP1B and LAP1C, normalized versus β-actin in individual 1´s dermal fibroblasts and a control sample (control 1). Note the absence of both LAP1 isoforms in individual 1´s dermal fibroblasts. On the right side the sizes according to the PageRuler Plus Prestained Protein Ladder are shown.

**Fig. S4.** **Quantification of lamin B1 protein levels (shown in Fig. 3c).**

**
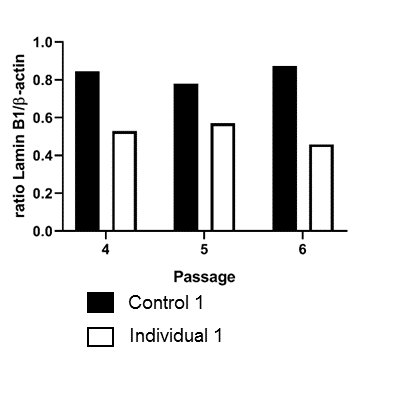
**

Quantification of protein analysis in three subsequent fibroblast passages (P4-6) of lamin B1 normalized versus β-actin in individual 1´s dermal fibroblasts and a control sample (control 1). Note that the corresponding blot is shown in Fig. 3c.

**Fig. S5. Reduced expression of lamins A and C in fibroblasts of individual 1.**


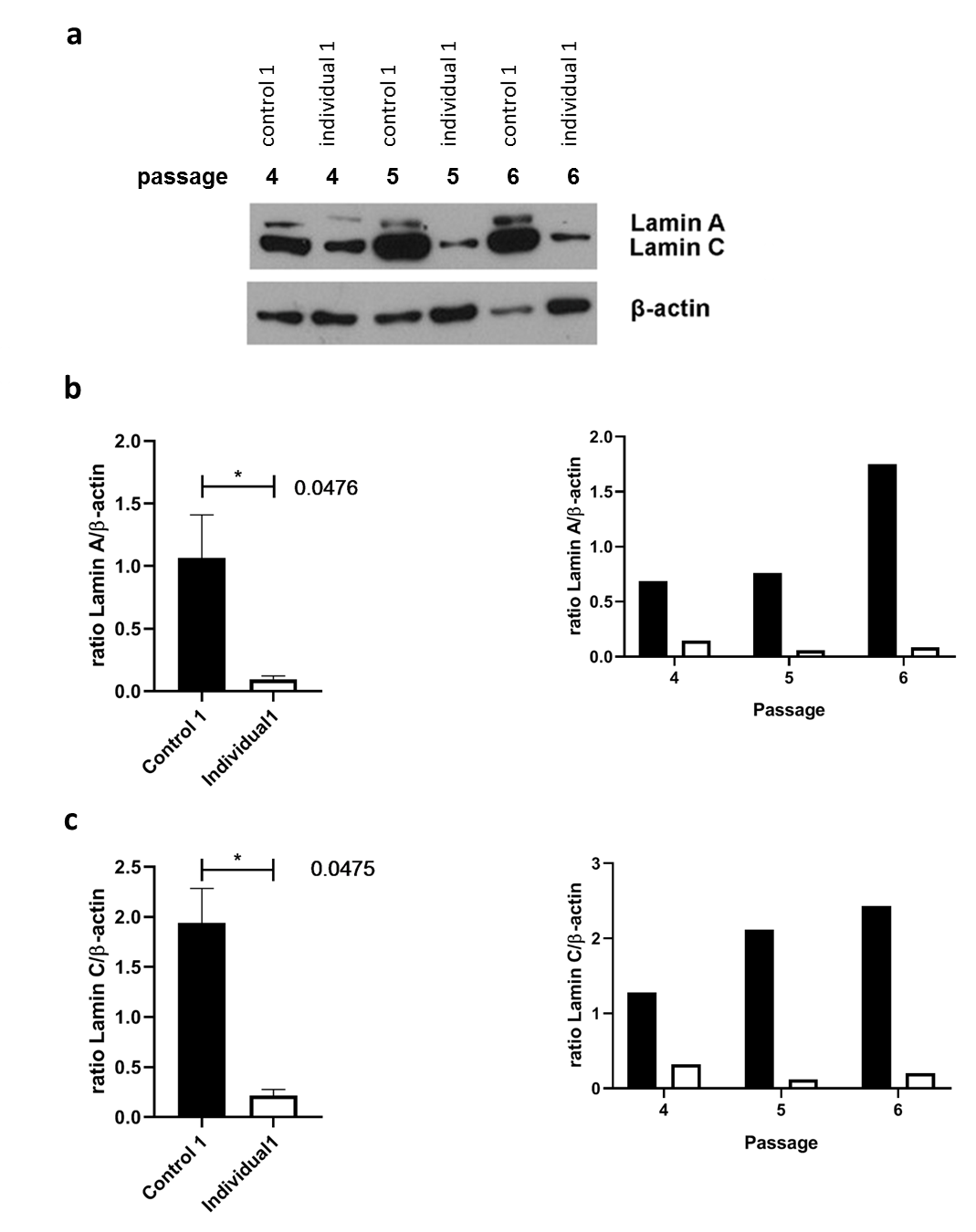


Protein analysis in three subsequent fibroblast passages (P4-6) of of lamin A and C normalized versus β-actin in individual 1´s dermal fibroblasts and a control sample (control 1). Note the low lamin A and C levels relative to control sample, in all three passages (**a**). Quantification of data in (**a**), shown for Lamin A and C, respectively, as a summary of the results in three subsequent passages (left) and in every passage (right) (**b,c**).

**Fig. S6.** **Quantification of phosphorylated S6 kinase (p-S6k) levels (shown in Fig. 5a and b).**

**
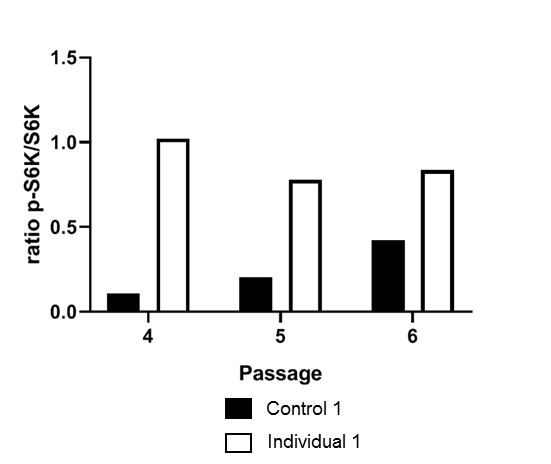
**

Quantification of phosphorylated S6 kinase (p-S6k) versus total S6 kinase (S6k) in each of the three subsequent fibroblast passages (P4-6), in individual 1´s dermal fibroblasts and a control sample (control 1). Note that the corresponding blot is shown in Fig. 5a.

**Fig. S7. Quantification of phosphorylated ERK1/2 (p-ERK1/2) levels (shown in Fig. 5d and 3).**

**
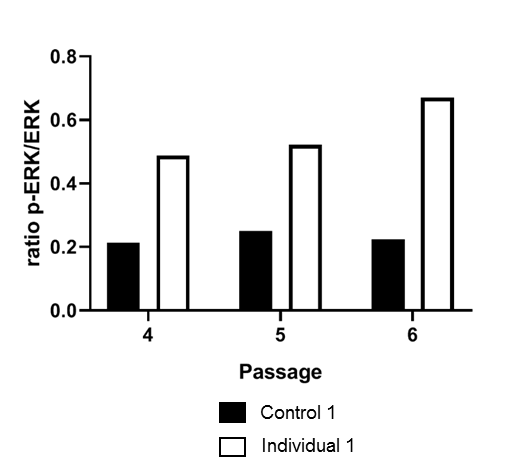
**

Quantification of phosphorylated ERK1/2 (p-ERK1/2) versus total ERK1/2 in each of the three subsequent fibroblast passages (P4-6), in individual 1´s dermal fibroblasts and a control sample (control 1). Note that the corresponding blot is shown in Fig. 5d.

**Fig. S8. Ectopic expression of wild-type TOR1AIP1 in fibroblasts from individual 1.**

**
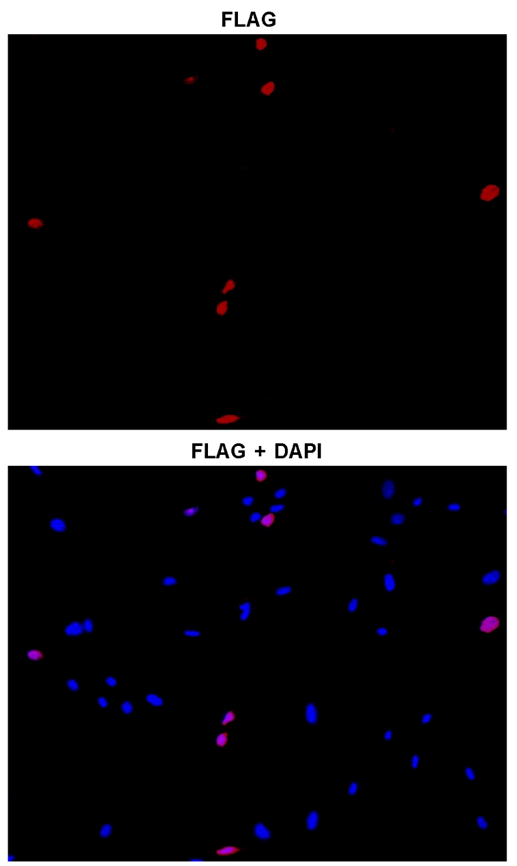
**

Representative image of conventional immunofluorescence microscopy with anti-FLAG antibody (red) and DAPI (blue) showing a transfection efficiency of around 20% in passage 8 of patient-derived fibroblasts transfected with 4 µg of wild-type Myc-Flag-tagged TOR1AIP1 plasmid. Image was taken using 20x magnification.

**Table S1.** Heterozygous variants identified in individual 1 by whole-exome sequencing (WES) that were previously not annotated in publicly available databases. All variants were validated and analyzed in parental DNA samples by Sanger sequencing.

| **gene** | **nucleotide change** | **protein change** | **patient** | **father** | **mother** |
| --- | --- | --- | --- | --- | --- |
| *AGFG1* | c.478C>G | p.L160V | heterozygous | heterozygous | WT |
| *AHNAK2* | c.9512C>T | p.P3171L | heterozygous | WT | **homozygous** |
| *CDH12* | c.2030C>T | p.A677V | heterozygous | heterozygous | WT |
| *CEP120* | c.2363A>G | p.N788S | heterozygous | heterozygous | WT |
| *CEP350* | c.2705C>T | p.S902F | heterozygous | WT | heterozygous |
| *CNIH2* | c.19G>T | p.A7S | heterozygous | WT | heterozygous |
| *CPM* | c.1246T>A | p.Y416N | heterozygous | WT | heterozygous |
| *CRY1* | c.722C>T | p.A241V | heterozygous | WT | heterozygous |
| *CSPG4* | c.6190_6191delGAinsTT | p.D2064F | heterozygous | heterozygous | WT |
| *DACT2* | c.1483A>G | p.K495E | heterozygous | heterozygous | WT |
| *DCST2* | c.240_245delCCT | p.L82del | heterozygous | heterozygous | WT |
| *DNAH14* | c.6016A>T | p.S2006C | heterozygous | heterozygous | WT |
| *EEF1B2* | c.181T>C | p.S61P | heterozygous | heterozygous | WT |
| *FAM20A* | c.23G>T | p.R8L | heterozygous | WT | heterozygous |
| *FZD9* | c.1751C>G | p.S584C | heterozygous | heterozygous | WT |
| *IGSF21* | c.827G>A | p.R276H | heterozygous | heterozygous | WT |
| *KIAA1549* | c.4682G>A | p.R1561Q | heterozygous | heterozygous | WT |
| *KNTC1* | c.6482C>T | p.T2161I | heterozygous | heterozygous | WT |
| *KRT10* | c.1456_1479delGGCCACGGCGGC | p.G490_G493del | heterozygous | WT | **homozygous** |
| *LAMB2* | c.1928A>G | p.Q643R | heterozygous | WT | heterozygous |
| *LRP5* | c.1700G>A | p.S567N | heterozygous | WT | heterozygous |
| *MCF2L* | c.2695A>T | p.T899S | heterozygous | heterozygous | WT |
| *MDGA1* | c.1759_1764delGTT | p.V588del | heterozygous | WT | heterozygous |
| *METTL21B* | c.664G>A | p.E222K | heterozygous | heterozygous | WT |
| *MYO1A* | c.110_112delT | p.Y38Ifs*8 | heterozygous | heterozygous | WT |
| *PHF7* | c.508C>G | p.Q170E | heterozygous | heterozygous | WT |
| *PI16* | c.827T>C | p.M276T | heterozygous | WT | heterozygous |
| *PPHLN1* | c.1268delT | p.I423Tfs*30 | heterozygous | heterozygous | WT |
| *PPP1R37* | c.1549G>A | p.E517K | heterozygous | WT | heterozygous |
| *RAPGEFL1* | c.596T>C | p.V199A | heterozygous | heterozygous | WT |
| *RIN1* | c.1078C>A | p.P360T | heterozygous | heterozygous | WT |
| *RXFP1* | c.1651G>A | p.E551K | heterozygous | WT | heterozygous |
| *SCNN1D* | c.464+5C>T |  | heterozygous | WT | heterozygous |
| *SLC7A6* | c.73_78delGAA | p.E26del | heterozygous | heterozygous | WT |
| *SOX4* | c.1155_1163delCTC | p.S388del | heterozygous | WT | heterozygous |
| *SPINK5* | c.2487_2490insGAGC | p.N831Efs*15 | heterozygous | WT | heterozygous |
| *SYNE2* | c.9520C>A | p.Q3174K | heterozygous | heterozygous | WT |
| *TBX18* | c.353C>T | p.P118L | heterozygous | WT | heterozygous |
| *TGM7* | c.1208A>G | p.D403G | heterozygous | WT | heterozygous |
| *TMEM25* | c.424G>T | p.A142S | heterozygous | WT | heterozygous |
| *TRIM50* | c.482G>A | p.R161Q | heterozygous | WT | heterozygous |
| *VAPB* | c.474_479delTTC | p.S160del | heterozygous | WT | heterozygous |

**Table S2.** Putative biallelic variants with minor allele frequencies (MAF) < 0.01 identified in individual 1. All variants were validated and analyzed in parental DNA samples by Sanger sequencing.

| **Gene** | **Nucleotide change** | **Protein change** | **patient** | **father** | **mother** |
| --- | --- | --- | --- | --- | --- |
| ***TOR1AIP1*** | **c.1331G>C** | **p.R444P** | **heterozygous** | **WT** | **heterozygous** |
| ***TOR1AIP1*** | **c.945_948delCAGT** | **p.Q315Hfs*9** | **heterozygous** | **heteroyzgous** | **WT** |
| *GMPPA* | c.250C>A | p.Q84K | heteroyzgous | heteroyzgous | WT |
| *GMPPA* | c.280G>T | p.G94C | heteroyzgous | heteroyzgous | WT |
